# Considering humans as habitat reveals evidence of successional disease ecology among human pathogens

**DOI:** 10.1371/journal.pbio.3001770

**Published:** 2022-09-12

**Authors:** Nina H. Fefferman, Charles A. Price, Oliver C. Stringham

**Affiliations:** 1 Ecology and Evolutionary Biology, University of Tennessee, Knoxville, Tennessee, United States of America; 2 National Institute of Mathematical and Biological Synthesis, University of Tennessee, Knoxville, Tennessee, United States of America; 3 Ecology, Evolution, and Natural Resources, Rutgers University, New Brunswick, New Jersey, United States of America; 4 The University of Adelaide, Adelaide, Australia; New York University School of Medicine, UNITED STATES

## Abstract

The realization that ecological principles play an important role in infectious disease dynamics has led to a renaissance in epidemiological theory. Ideas from ecological succession theory have begun to inform an understanding of the relationship between the individual microbiome and health but have not yet been applied to investigate broader, population-level epidemiological dynamics. We consider human hosts as habitat and apply ideas from succession to immune memory and multi-pathogen dynamics in populations. We demonstrate that ecologically meaningful life history characteristics of pathogens and parasites, rather than epidemiological features alone, are likely to play a meaningful role in determining the age at which people have the greatest probability of being infected. Our results indicate the potential importance of microbiome succession in determining disease incidence and highlight the need to explore how pathogen life history traits and host ecology influence successional dynamics. We conclude by exploring some of the implications that inclusion of successional theory might have for understanding the ecology of diseases and their hosts.

## Abstract 2

This study explores the analogy between ecological succession in terrestrial ecosystems and infections in a human-host landscape over time, showing how the ecosystem of long-term multi-pathogen dynamics within and among hosts may be a critical missing consideration in understanding epidemiology.

## Introduction

Research into the epidemiology of infectious diseases has benefited greatly from the realization that pathogens and parasites have their own natural ecology (as reviewed and synthesized in [1–3]). These insights have spanned the range from basic theory (e.g., [4,5]) to applied management strategies (e.g., [6–8]). Incorporating the effects of competition among strains of the same disease, within and between hosts, has increased our understanding of spatiotemporal patterns in outbreaks (e.g., [9,10]). Similarly, many studies have examined the impact of ecological dynamics among multiple hosts susceptible to a single parasite/pathogen (e.g., [11]). Valuable insights have arisen from theories that integrate concepts from biodiversity, conservation biology, and disease ecology (e.g., [3,12–15]). More recently, there has been an expansion of these perspectives to multiple diseases circulating among multiple host species (cf., [16]), leading to a deeper understanding of the ecological, epidemiological, and evolutionary dynamics of disease at the ecosystem level (cf., [17,18]). While most frequently focused on parasites, analyses developed for food webs have also yielded profound insight into disease ecosystems (e.g., [12,19–21]) and suggested ways to discover how targeted management might interrupt interspecies disease transmission networks (e.g., [22]). Methods from the study of metapopulations have also been leveraged with great success, looking at disease outbreaks among mostly isolated populations (re)introduced by migration and/or travel (e.g., [23–25]). Together, these perspectives have provided a more diverse and powerful toolkit for characterizing and predicting disease dynamics.

While epidemiological research has already benefited in many ways, we have far from exhausted the potential of ecological theory to inform our understanding of infectious diseases. Many of the insights from community ecology are only now beginning to be discussed in application to epidemiology (cf., [26]). Invasion ecology (the study of when species new to a particular area or habitat can establish and spread successfully; cf., [27]) has thus far been primarily applied to introduced vectors of infection [28,29], but there are nevertheless clear parallels to every facet of outbreak (re)emergence that have begun to be explored [30–32]. In fact, mathematical models from epidemiology that explicitly incorporate both diffusive spread of infection among local contacts and the impact of rare, long-distance dispersal into previously unaffected communities, exactly mirror the concepts from invasion ecology of novel introduction, initial establishment, and subsequent spread. These ideas have been well studied in many disease systems, but only recently have studies begun to incorporate insights from ecological theory in their approaches (e.g., [31,33]).

One of the most underappreciated concepts from ecology that can inform epidemiology is that of succession [34–37]. At their most basic, theories of ecological succession predict directional change in community composition over time. Embedded in community succession are assembly patterns where pioneer species from the regional species pool are established based on their life history characteristics. Pioneer species can in turn modify niche space within the community to facilitate the establishment of secondary successional species with different life history traits [38–43]. In this way, existing species assemblages facilitate the introduction and/or growth of some species while impeding the introduction and/or growth of others. This impedance can be either by direct competition for resources (niche preemption) or else by degrading the suitability of the habitat in other ways (niche modification). Facilitation can occur through mutualistic interactions or through habitat enhancement. When the growth of extant species with similar life history characteristics are reduced to the point that they are replaced entirely by a set of species with different life history characteristics, this is called species turnover.

While certainly not the only force at work in shaping the assembly of species within a community, ideas from succession theory have greatly enhanced our understanding of the community ecology of diseases. The concept of species turnover has been applied successfully to understand particular medical conditions influenced by an individual’s microbiome (cf., [44]). Studies have provided both theoretical insight and clinical recommendations in application to gut [45,46], vaginal [47], oral [48], nasal [49], and dermal [50] microbiota, exploring the transition from established but harmless colonization, to clinical pathology, and what might be done to mitigate medical risks via restoration of a healthy microbiome [51,52].

An understanding of ecological theory is not required to determine the balance of microbes that support healthy human function. However, isolated studies of disease pathology lack the benefit of comparing patterns across physiological systems [53,54]. Without such cross-system analyses, it would be difficult to make predictions for which classes of extant microbes might become pathogenic under perturbation (e.g., antibiotic therapy), or which types of microbes might be opportunistic, invading only when the opportunity is created by disruption to normal microbiotic systems (e.g., dietary shifts). An ecological perspective that embraces historical contingency aids the design of interventions that target function of the human microbiome at the community level.

While this recent body of work has enabled greater use of ecological perspectives for within-host microbial communities, it has yet to be applied broadly to population-level epidemiology. Unfortunately, the link between microbiome succession and the epidemiology of infectious diseases in human populations is not necessarily straightforward. Some studies of metapopulation disease dynamics have incorporated spatial and temporal aspects of spread among (populations of) hosts as habitat patches, but it is challenging to characterize the successional stage of a human host microbiome. Traditional ideas of succession consider “early stage” environments that have recently been disturbed in some manner (fire, flooding, etc.) or “later stages” of succession (defined by the set of species present). The human microbiome is comprised not only of its microbial community, but our immune system and previous exposures to infection also impact “habitat quality” for any new pathogen or parasite. In other words, the habitat suitability and invasibility of the host environment for each introduced pathogen will be due in part to the individual host’s immune function, which will have been shaped by the progression of disease exposures they have experienced, and in part due to the community of hosts who have potentially experienced different infections but have perhaps influenced their immune function in similar ways. The progression of pathogen exposures may therefore be considered a successional process. (Note that there are, of course, other factors that influence the ability of a pathogen to infect a host, including but not limited to host nutritional status, age, etc.) Although not usually considered through the lens of succession, the interactions between the rate of recruitment of novel susceptibles (e.g., via birth rate) and the periodicity of outbreaks [55] already demonstrates that humans as habitat are changed by their life history of disease exposure in ways that impact population-level disease dynamics.

A population of hosts therefore functions as a collection of interacting individuals each with its own distinct immune memory, together constituting a habitat patch. Within this environment infectious diseases have the potential to interact directly (via cocirculation within a population and/or coinfection within an individual host) or indirectly (via the immune system of potential hosts) with all other pathogens and parasites. The influence of immune memory on habitat suitability means that not only is there niche preemption and/or modification from cocirculating pathogens, but there is the potential for influence from every pathogen and parasite that has circulated in the host population during the collective duration of immune memory (see S1 Text). The host population’s collective microbiome can impede the success of a novel infection via competition for within-host resources or through habitat degradation due to prior exposure to a pathogen yielding cross-protective immunity (cf., [56]). Conversely, there is also clear evidence of at least 2 separate mechanisms for successional facilitation among pathogens. The first is when the host’s immune system is altered by active infection, allowing opportunistic secondary infection from pathogens/parasites that would otherwise be easily thwarted by normal host immune function (cf., [57–60]). The second, only recently described mechanism is that of immune amnesia, in which infection disrupts previously gained immune memory [61,62]. This latter mechanism effectively restores the hosts as accessible habitat for diseases that had previously been introduced and would have otherwise had to wait for demographic/generational replacement to yield enough susceptible individuals to permit successful, ongoing transmission [55].

Viewing memory-influenced hosts-as-habitat patches through the lens of successional ecology enables testable predictions about the nature of interactions among human diseases. Just as certain life history characteristics could enable a shrub to establish in a grass meadow, so too should diseases with particular sets of etiological traits be able to exploit niches created by the history of niche modification that results from previous pathogen exposure. The epidemiological equivalent of habitat is the host’s microbiome, inclusive of the host’s immune memory, shaped by the progression of previous disease exposures. Therefore, where ecological theory discusses time since the creation or disturbance of a given habitat, successional epidemiology must consider the age of the host within each generation and their previous disease exposure. Just as plants that share life history traits might establish in habitat patches at about the same time since the last disturbance of the patch, we hypothesize that diseases that share life history traits may tend to infect the same demographic life stages of their hosts within a population (henceforth referred to as “Age of Greatest Prevalence”).

Our formulation of this hypothesis shares features with early ideas about ecological succession, which were initially formulated as qualitative descriptions of sets of plant life history traits thought to influence succession, such as seed dispersal distance, vegetative growth rate, and shade tolerance [36]. Some quantitative models have been proposed to explore the mechanisms and patterns that might drive succession, but the characterizations and predictions are, first and foremost, qualitative in nature across systems. In keeping with this research, we form the following qualitative hypothesis: If there is ecological succession in infectious diseases in the human-host-habitat, there should be distinct sets of life history traits of pathogens that influence the temporal sequence of disease incidence in host populations. In other words, we should be able to identify sets of traits of parasites or pathogens that correlate directly to a sequence of infections that should be consistent with the general order in which people are exposed to/infected by these diseases throughout their lives. It is important to note that this hypothesis is complementary, rather than in conflict with those that posit population-level patterns in disease spread; the ecological habitat for pathogens is therefore comprised of the aggregate community of host individuals who have been affected over their individual lives by the pathogens to which they have been exposed.

This hypothesis relies on the idea of habitat quality for an infection being determined at both an “individual host” level and a “community of hosts” level. Extending the analogy with successional ecology of plant ecosystems: A particular patch may never have a specific species of grass grow within it but is nevertheless primed by the growing of “some grasses” for the arrival of “a shrub”. Similarly, the growth of taller plants provides the opportunity for shade-tolerant species to grow; shade is a property of the community rather than the result of the presence of a particular taller plant or species. In this way, while hosts may experience different infections, if the pathogens serve the same successional role, they may together increase the probability of success for establishment following introduction of the next infection (whether endemic or epidemic) into the host population. This then increases the probability for each individual host to experience “an infection of the next successional type” but does not specifically imply that the host will catch any particular pathogen. That increased probability also improves the likelihood for new pathogens of the next successional stage to be able to establish, circulate, and infect other hosts (thereby altering their own host habitat again).

To test this hypothesis, we identify 6 candidate life history traits that we hypothesize will influence the degree to which a list of well-known diseases successfully infects human populations over host lifetimes. To summarize the collective effects of these traits we introduce a qualitative “Successional Score” for each pathogen and test its ability to predict the average age of humans experiencing the greatest prevalence of reported infection (see Methods below).

## Results

**Fig 1:**
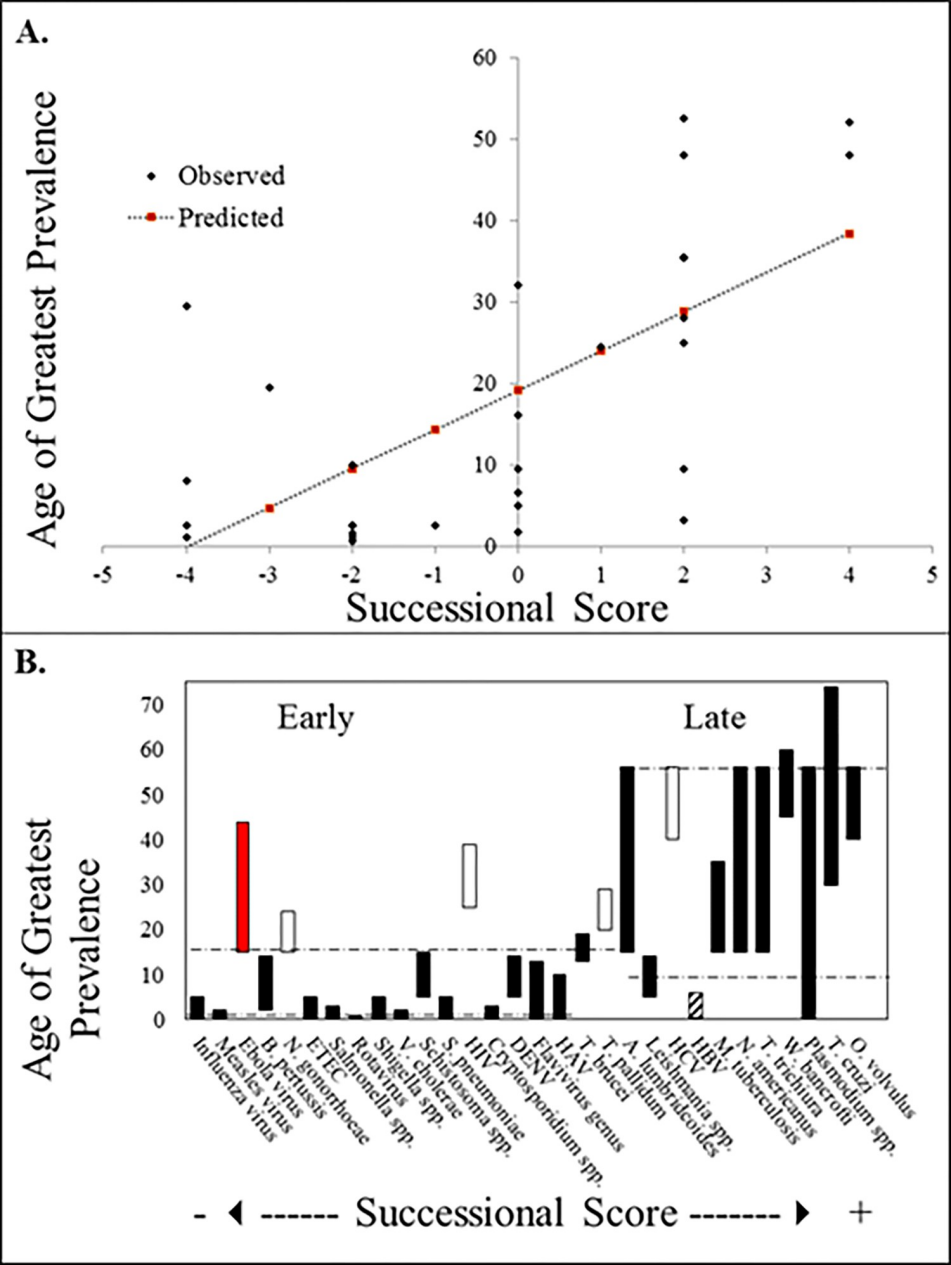
Analyses of the correlation between Successional Score and Age of Greatest Prevalence. In Panel A [F(1,28) = 21.85, *p* < 0.001 with an R^2^ of 0.44], note the classification of Age of Greatest Prevalence into “Early” and “Late” by Successional Score alone (with a break-point of ≥2) (B) [Z-Score = −3.59, *p* < 0.001]. (B) Unfilled boxes represent sexually transmitted infections, except hepatitis B, represented by the striped box. The red box represents Ebola. Dashed horizontal lines provide mean maximum and minimum ages across pathogens in the “Early” and “Late” classification, respectively. The data underlying this figure can be found in S1 Data.

The Successional Scores produced for our 30 pathogens were not only significantly correlated with particular demographic categories in the age of greatest prevalence for each disease (Fig 1A), but the classification of age groups based only on the Successional Score was also found to be meaningful (Fig 1B). These results would be equally meaningful with a reversed trend; support of a successional hypothesis relies on observable ages-as-stages resulting from common life history traits, rather than on which suites of traits yield which particular ages of greatest prevalence.

These results are clearly consistent with the hypothesis that some form of successional progression is observable in the disease ecology occupying the human-host-habitat. While the particular life history traits of the pathogens and parasites tested already seem to support this theory, the qualitative nature of the classification does not at all rule out the existence of a better, more predictive set of traits for analysis in the future. Future efforts gathering more life history data across a wider array of disease taxa will help to determine which traits are most informative in understanding the influence of historical contingency and successional processes in the incidence and timing of human diseases.

**Fig 2:**
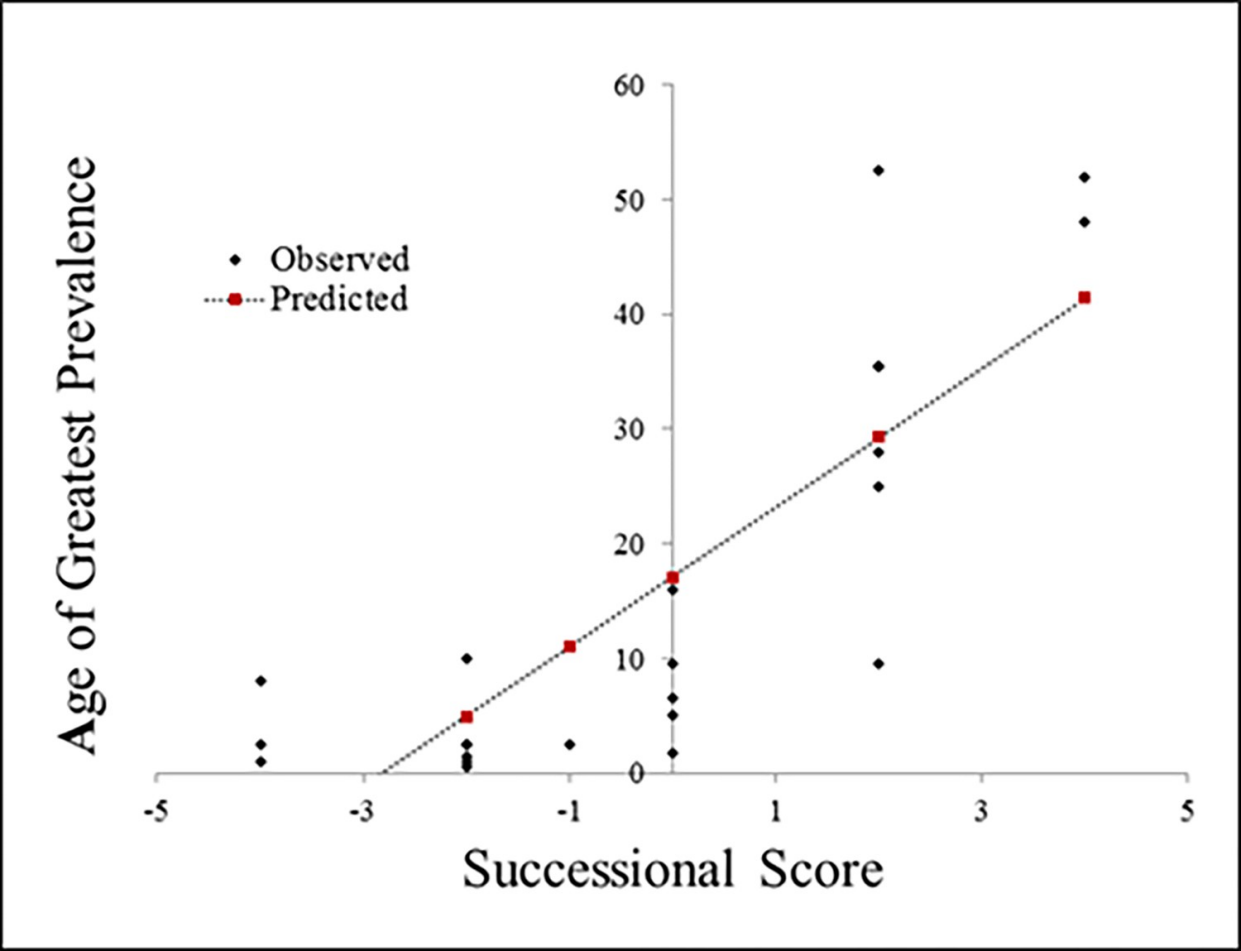
Correlation between Successional Score and Age of Greatest Prevalence. After omitting sexually transmitted infections and Ebola from the analysis, the observed R^2^ value increases [F (1, 22) = 46.49, *p* < 0.00001 with an R^2^ of 0.68]. The data underlying this figure can be found in S1 Data.

While the results for our full set of diseases are encouraging, patterns among the regression residuals are of interest and potential importance. Five of the 10 diseases with the largest residuals (absolute value) are sexually transmitted infections. Regardless of whether their etiological features suggest that they should be prevalent among younger age groups, these diseases are functionally constrained by the nature of their mechanism of transmission to greater prevalence among sexually active age groups (with the further exception of hepatitis B, which, despite normally being considered a sexually transmitted illness, demonstrated high rates among children in developing nations without consistent access to vaccination [63]). The disease with the largest residual is Ebola, a disease so recently emerged that our current estimation of the age of greatest prevalence is driven by small samples and early post-emergence transient dynamics rather than long-term successional behavior. If this is the case, an intriguing and potentially important prediction from this early investigation is that Ebola may actually be a disease of younger ages than has been observed thus far. If true, this has broad implications for targeting vaccination strategies and long-term public health interventions. When the regression analysis was performed again, excluding the sexually transmitted illnesses and the newly emerged Ebola, the correlation grew stronger (Fig 2). The significant correlations observed both with and without sexually transmitted diseases (and Ebola) provide strong support for the idea that succession theory can begin to inform discussions of disease ecology, even at this fairly coarse and qualitative scale.

To test whether other potential scores from the same combination of features could also predict patterns in prevalence, we applied the same method to all potential combinations (Note: The proposed score was hypothesized first, based on ecological principles and was not chosen due to its predictive power.) In contrast to the results from the proposed actual Successional Score, the best alternative combination of features produced a score that made no significant prediction (*F* (1, 28) = 2.47, *p >* 0.1 with an *R*^2^ of 0.08).

**Fig 3:**
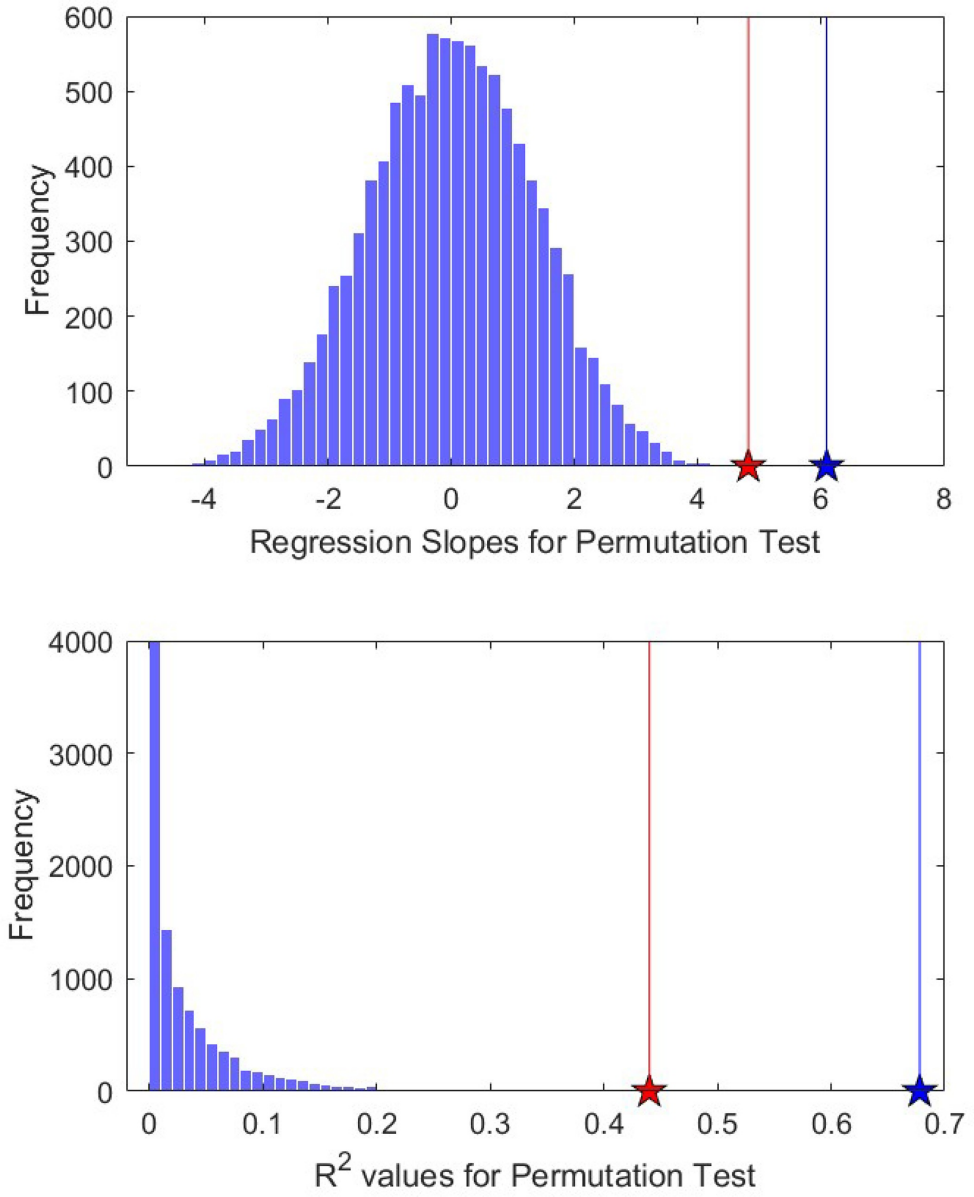
**Frequency distribution of permutation test regression slopes (Panel A) and *R***^***2***^ **values (Panel B)** (see Methods). Note observed slope and *R*^*2*^ value for all data (blue star and line) and for data without sexually transmitted diseases and Ebola (red star and line) fall well outside of permutation test distributions. The data underlying this figure can be found in S2 Data.

As seen in Fig 3, our observed slope and *R*^*2*^ values for all data (blue star and line) and data without sexually transmitted diseases and Ebola (red star and line) are both well outside the permutation test generated distributions.

Both of these statistical approaches clearly demonstrate that the classification of similar etiological characteristics of diseases are not trivially correlated to age of greatest prevalence. This shows that not only is our hypothesis falsifiable, but that it is reasonable to infer meaning from these patterns. This finding also suggests that, since our feature set does not provide a direct, natural analogue to the sets of traits that govern plant/animal succession, the nature of the ecology of pathogens and parasites may require separate theories about reproductive strategies that go beyond the direct evolution of virulence (as has been done in some cases already; [64] and [65] among others).

As with the tested alternative combination of features, considering the reproductive number, *R*_0_, for each pathogen as a potential correlate for age of greatest prevalence yielded no significant inference (*F* (1, 26) = 0.04, *p >* 0.1 with an *R*^2^ of 0.001; malaria and ETEC were excluded due to complications in estimating a meaningful *R*_0_ value). (It should be noted that, while epidemiological theory suggests that the reproductive number of a disease should be inversely correlated with the age of first infection, many of the diseases here discussed do not generate life-long immunity, and thus the age of first infection is substantially different from the age experiencing greatest disease prevalence.)

While not perfectly predictive, the proposed Successional Score was able to offer significant insight into the age of greatest prevalence. An important future direction will be to perform these same analyses within developed nations, in a modern healthcare setting, and determine whether or not the same etiological features and assumed alignments in features will yield similarly meaningful sets of successional traits. Determination of the best, most predictive, suite of traits for predicting disease succession may itself provide otherwise inaccessible insight into the community dynamics among infectious diseases.

## Discussion

Our results demonstrate that the community dynamics of infectious diseases should be considered more broadly in discussions of disease and human health than simply within the context of individual microbiomes. The expansion of epidemiology to include successional dynamics not only enriches our understanding of basic theory but leads to a variety of practical implications. Just as the realization that disturbance to a patient’s microbiome can influence their susceptibility to developing pathological illness from infections (e.g., *Clostridium difficile*) have altered clinical practice and case management, public health planning may benefit from being informed by an understanding of the likely behavior of outbreaks of different infections given the immunological history (i.e., successional stage) of the population at risk. Instead of restricting consideration to prior outbreaks of diseases only known to cause some level of cross-protective immunity, successional theory suggests that the entire immunological profile of the population is likely to affect the relative success of each attempted reinvasion/emergence, even of the same pathogen in the same population.

While there are many possible potential mechanisms that could contribute to this observed ecological phenomenon, the most likely are found in the life history progression of individual immunological function and how that then scales to a population-level habitat suitability for each next pathogen invader. Adaptive immune function has long been understood to offer some cross-reactive protection to novel strains of a circulating pathogen [66,67] and has more recently been understood to be compromised by certain infections, even going so far as to “reset” vulnerability to unrelated pathogens, so called immune amnesia [62,68]. There have also been intriguing studies highlighting the potential for both short- and long-term changes in innate immune protection following particular infections [69,70]. There has even been some evidence of direct epigenetic modulation of immune function following microbial exposures [71]. While it is therefore likely too early to propose a specific and unique mechanism for the observed successional dynamics in pathogens and parasites, there are ample potential pathways that make hypothesizing such an effect immunologically plausible.

Of course, ours is not the first study to suggest a causal link between host life history and the timing of infection from specific pathogens. Anderson and May provided a simple calculation for the expected age of first infection for pathogens that cause long-standing immunity [72]. We do not mean to suggest that successional dynamics alone shape these outcomes any more than we suggest that life history and immunity are solely responsible. Were immunity and susceptible recruitment the only driving factors in age of infection, we would expect the same pathogen to demonstrate different age ranges in populations with different immunocompetence, birth rates, and immigration patterns; clearly this is not the case for all infections.

Testing the successional hypothesis beyond the phenomenological evidence provided here is not a trivial task. An ideal empirical study would rely on comparative time series data for outbreaks of pathogens across populations that experience different patterns of exposure. An ideal dataset might come from a single population that then functionally fissions (whether by migration or cultural practice), with the different surviving coherent subpopulations then experiencing exposure to different types of pathogens. By then contrasting the average age of greatest prevalence for infections that affected the different subpopulations under their now different immunological trajectories, we could begin to tease apart whether aggregate immunological life history truly impacted population-level susceptibility to outbreaks of particular pathogen types. Of course, this proposed example still focuses on human infections. For practical reasons, collecting age-specific information about infections in wildlife populations is not standard practice, but we would hypothesize the same dynamics should be at work, at least among pathogens in vertebrate hosts. Mounting such lab or field-based studies would pose their own challenges but could also provide better evidence/understanding of successional dynamics as a meaningful component of disease ecology.

Once the features and mechanisms that determine successional stages for pathogens and parasites are better studied and more thoroughly understood, we will be able to improve our estimation of risks from (re)emerging epidemics. Analysis of successional dynamics may also allow us to understand the differences between short-term, transient dynamics of newly emerging pathogens, and pathogens whose etiology has been newly altered by advances in medical practice, and the long-term, stable dynamics of diseases that are either endemic or consistently reemerge into the same populations. This would enable predictions about the consistency of behavior of outbreaks for the same pathogen over time and across populations, improving our ability to plan effective public health interventions.

As a number of recent disease outbreaks (SARS, H1N1 2009, Ebola, Zika, and COVID-19) have evidenced, our models for risk assessment develop in real time with the expansion of each new epidemic. Epidemiological rates, such as socially mediated contact-based transmissibility, must be estimated in each affected population, either by direct observation and measurement or else by fitting epidemic models to observed case incidence curves to find parameters that yield the best agreement. While such approaches will remain important, if we can leverage ideas from succession, with only a very basic understanding of etiological features of new (or newly reemerging) diseases, we can consider the current successional stage of each population/region under threat and make meaningful complimentary predictions about their susceptibility to widespread outbreak from a disease of the relevant type. For example, we may eventually be able to identify which populations are most at risk from a particular type of new disease before it emerges and take medical/public health steps to prepare for outbreaks of that type or at least to enact targeted surveillance in those communities for these “high-risk” outbreak types. Of course, as in ecology, successional stage is not the only driver of success for introduced species, so we envision this as a complimentary approach to broad surveillance strategies.

Extending successional ecological theory beyond the microbiome to the macroscale of public health, we may be able to identify types of perturbations to the host-disease system that enable the emergence of outbreaks. Just as *C*. *difficile* opportunistically exploits perturbation in individual host microbial communities to cause disease, we might find that seemingly stochastic emergence of zoonoses are actually the result of particular types of perturbations in the health ecosystem. Perhaps a vaccinated cohort reaches a particular density and demographic distribution within the broader population, or a new antibiotic diminishes the circulation of an entire class of competitor pathogens, and the resulting opportunity is just waiting for a pathogen with the right life history characteristics (whether newly mutated or merely newly reintroduced) to arrive and cause an outbreak it could not have caused only a few years before. This perspective adds a new lens to quantitative risk estimation of the epidemic potential for both entirely novel diseases or newly mutated strains of existing pathogens, a field that has important implications for multinational coordination efforts in pandemic preparedness and response [73,74].

Though the work presented here is merely a first step, these explorations demonstrate how a successional perspective on the behavior of infectious diseases may be able to meaningfully contribute to strategies for outbreak management and public health preparedness. Recent advances in our understanding of single-disease systems have reached sufficient maturity to enable their integration toward a broader, more unified theory of disease ecosystems. While only a first set of investigations, the success we have demonstrated with limited sets of disease life history features challenges us to develop theories about how these features interact to create a disease successional ecology and which other features might also be important in shaping theses dynamics. We anticipate with excitement the insights that an epidemiological analysis of the successional disease ecology of human health will provide.

## Materials and methods

Initial formulations of successional theories (in plant communities [36]) relied on understanding how similar traits among species influenced the successional stage (also sometimes called “seral stage”) in which they were found in greatest abundance. Particular species assemblages were then proposed to facilitate the invasion/establishment of subsequent species cohorts that would gradually replace them and in turn pave the way for yet other species to establish. In order to apply this perspective to infectious diseases, we seek to discover/describe sets of etiological traits that cause similar dynamics (see S1 Table). In considering which suite of candidate traits might be important in disease succession, many potentially important life history traits were inaccessible due to a lack of data collected about them in ways consistent enough to be compared across pathogens. For example, it might be important to know the average duration of natural immunity; however, due to confounding influences from herd immunity, this is unknown for many diseases. As a result, our analyses focus on 6 features that were hypothesized to be of potential importance to disease succession, due to their similarity to features important in plant systems, and also their availability in ways that allowed meaningful comparison across a sufficiency of diseases (at least 20 out of the 30 diseases included in analysis). These features were (1) the duration of the incubation period; (2) the duration of the infectious period; (3) the viability of the pathogen outside of its human host; (4) the physical distance over which the disease is capable of being transmitted between hosts (e.g., fluid contact versus droplet range versus aerosolized range); (5) how often the disease is opportunistic, exploiting the presence of another pathogen/parasite as the means by which to successfully infect a new host; and (6) the mutability of the disease (the rates of antigenic drift or emergence of antibiotic resistance, etc.). While most of these features were chosen based on analogues in the plant succession literature [35], the suite of features that correlate most strongly with common ages of greatest disease prevalence will inform hypotheses for how diseases might interact ecologically to shape disease succession.

Medical intervention is another possible confounding influence on our ability to discover successional patterns across infectious diseases. Any investigations into the validity of the successional hypothesis needs to rely on information about disease characteristics and medical practices that are consistent across diseases. As a result, for this initial investigation, we restricted our analyses (wherever possible) to disease burdens in developing nations with similarly limited access to healthcare. This is not to suggest that ubiquitous access to medicine would invalidate the hypotheses of disease succession, merely that medical aid would be very likely to alter successional patterns, and therefore, it is important to look for patterns first within regions that share similar levels of access to healthcare and medical interventions.

Within these structured assumptions and parameters, we then performed a literature review searching PubMed and Google Scholar for “etiology” and each pathogen to find estimates of our 6 features for each of 30 infectious pathogens (see S2 Table), chosen specifically to include epidemiological diversity in both mechanisms of transmission (fomite, droplet, aerosol, etc.) and pathogen/parasite taxa (bacteria, viruses, parasites, etc.). Because of the great diversity in characteristics among the features, across these diseases, we considered these descriptions as categorical ranks (as is traditional in plant successional models, for example, in comparing seed dispersal mechanisms and/or vegetative regeneration capability). Once considered as categorical ranks (Tables as defined in S3 and S4 Tables to yield scores S5 Table), we computed the median rank represented for each feature. While the actual median test has fallen out of favor as a statistical test of significance due to low power, the technique still provides a useful method for producing an analyzable scoring system from categorically ranked data. Thus, we assigned to each disease feature a feature score of ±1 based solely on whether it was above or below the median rank for that feature and then summed the feature score over the 6 features to produce our Successional Score for each disease. This nonparametric method was chosen to minimize the potential for synergistic effects in the magnitude of influence among features. To determine whether or not our Successional Score had yielded a valid correlative model of the age of greatest prevalence for each disease, we performed both linear regression on the pairwise values, and also a Mann–Whitney U test on the mean ages for diseases divided only by have a Successional Score greater or equal to, versus less than, zero.

To ensure that the hypothesis is, in fact, falsifiable and it is not simply the case that any consistent description of infectious diseases by etiological characteristics would produce meaningful correlation with the age of greatest prevalence, we also performed the same analysis on alternative versions of the Successional Score, in which different sets of traits were assumed to be aligned to produce consistent successional behaviors (e.g., instead of assuming that shorter than average viable transmission distance should act synergistically with high mutability, we also tested a score based on assuming the opposite direction of correlation). Again, since the feature set tested was merely a first hypothesis, we also performed the same analysis for all subsets of the feature set to test which combination of our 6 features might produce a successional score that correlated most strongly with the age of greatest prevalence (for further discussion and results from those tests, please see S2 Text). Lastly, we tested the correlation of age of greatest prevalence with the reproductive value, *R*_0_, for each disease to ensure that our etiological characteristics were not simply epidemiologically tautological (i.e., that our Successional Score was not simply an inelegant proxy for an already well-studied epidemiological metric).

As a further test to determine whether our results could have been obtained by chance, we employed a permutation testing approach, randomly assigning our observed successional scores to age of greatest prevalence values. This process was repeated 10,000 times to generate distributions of slopes and R^2^ values from least squares regression fits to each randomized set.

## Supporting information

S1 Text Individuals to populations—An explanation of how the ideas discussed rely on both individual host- and population epidemiological-level dynamics. (DOCX)

S2 Text Feature sets—An explanation and analysis of alternative combinations of subsets of features and their associated successional scores. (DOCX)

S1 Table Features of successional importance in plants and their hypothesized analogue for pathogens—A table presenting our proposed analogies between traditional features considered in ecological succession of plants and etiological and epidemiological features of pathogens. (DOCX)

S2 Table Citations for data used to calculate Successional Scores, Ages of Greatest Prevalence, and R0 values—A table presenting the literature from which the data used to assign features scores to each pathogen was drawn. (DOCX)

S3 Table Rank values for infectious period, incubation period, and duration of viability outside the host—A table presenting the bin duration for the temporal windows of features into scores. (DOCX)

S4 Table Rank values for transmission distance, opportunistic, and mutability—A table presenting the categorical designation for the nontemporal features. (DOCX)

S5 Table Categorical ranks for each disease used to calculate Successional Scores—A table presenting the rank designation assigned based on the literature and used to calculate the successional score. (DOCX)

S1 Data An excel spreadsheet of the feature rank scores for each disease. (XLSX)

S2 Data An excel spreadsheet of the data used to create Figs 1 and 2. (XLSX)
