# Supplementary material for: Considering humans as habitat reveals evidence of successional disease ecology among human pathogens
Source: PLoS Biol. 2022 Sep 12;20(9):e3001770. doi: 10.1371/journal.pbio.3001770 (PMC9467372; doi:10.1371/journal.pbio.3001770)
Supplement: S1 Text — (DOCX) [file pbio.3001770.s001.docx]

**S1 Text**

**Individuals to Populations.** While the immune system of each host will have been shaped by the complement of pathogens and parasites it has faced during its life, it would be a mistake to consider disease succession as a set of purely independent, parallel within-host dynamics occurring in each individual host. Although these effects are individually observable (cf. *C. difficile*; [1]), the predictions being made are not direct predictions solely about the order in which individual hosts should be susceptible to individual pathogens. Instead, these immune memory-based susceptibilities should influence the potential transmissibility of different diseases, meaning that the relationship between successional scores (and the features contributing to them) and the correlated demographic age of greatest prevalence could be expected to change over generations, as new hosts are born into populations with dynamic immune-demographies. As a result, successional effects are likely not only to operate at the level of a host, but may impact patterns of (re)emergence of diseases, and their spread, at the level of the population and over generational timescales.

**References**

1. Chang JY, Antonopoulos DA, Kalra A, Tonelli A, Khalife WT, Schmidt TM, et al. Decreased diversity of the fecal microbiome in recurrent Clostridium difficile—associated diarrhea. Journal of Infectious Diseases. 2008;197(3):435-8.
