# Supplementary material for: Considering humans as habitat reveals evidence of successional disease ecology among human pathogens
Source: PLoS Biol. 2022 Sep 12;20(9):e3001770. doi: 10.1371/journal.pbio.3001770 (PMC9467372; doi:10.1371/journal.pbio.3001770)
Supplement: S2 Text — (DOCX) [file pbio.3001770.s002.docx]

**S2 Text**

**Feature Sets.** Since the feature set we considered was created based on hypothesized analogies with traits important to plant succession, there was no guarantee that inclusion of each of our features would improve the power of the successional score to predict the age of greatest prevalence for each disease. To test this we performed the same analysis with every possible subset of features (including individual features), leading to 63 different analyzed potential feature sets defining 63 hypothesized successional scores. While the correlative fit did improve when omitting the “opportunistic” and “pathogen viability outside of the host” features (changing from a significant *R*^2^ of 0.445 to 0.490), this was driven entirely by a stronger correlation of “low successional score with Early age”. Unfortunately, this stronger early signal came at the expense of fit for late signal, meaning that although a low successional score did clearly indicate an Early disease, a high successional score could not differentiate between early or late diseases. Since the goal of a successional model is to provide accurate predictions of disease burdens based on ecological features, model selection should favor cases in which the most classifications of predicted age of greatest prevalence are assigned correctly by the hypothesized successional score. Failure to differentiate between early and late diseases among diseases with high successional scores are therefore less desirable. For the features considered, the best successional hypothesis was the one that included all of the considered features. It is of paramount importance to note that this does not imply that the considered features are the complete, final, or the best set of ecological features that could be used to understand successional epidemiology, merely that they are supported by the data as having some impact and the full set is the most effective set we have considered here. It is our hope that future endeavors to improve available data for different features and expand the number of diseases considered will enable better and better insight into successional features, just as decades of research has been published in refining insight into the importance of sets of ecological features in the succession of plants.
