# Supplementary material for: Considering humans as habitat reveals evidence of successional disease ecology among human pathogens
Source: PLoS Biol. 2022 Sep 12;20(9):e3001770. doi: 10.1371/journal.pbio.3001770 (PMC9467372; doi:10.1371/journal.pbio.3001770)
Supplement: S1 Table — (DOCX) [file pbio.3001770.s003.docx]

**S1 Table. Features of Successional Importance in Plants and their Hypothesized Analogue for Pathogens**

| **Plants** | **Pathogens** |
| --- | --- |
| Growth rate | Incubation period |
| Lifespan | Infectious period |
| Seed viability | Pathogen viability outside host |
| Dispersal distance | Transmission distance |
| Shade tolerance | Opportunism |
| Structural strength | Mutability |
