# Supplementary material for: Considering humans as habitat reveals evidence of successional disease ecology among human pathogens
Source: PLoS Biol. 2022 Sep 12;20(9):e3001770. doi: 10.1371/journal.pbio.3001770 (PMC9467372; doi:10.1371/journal.pbio.3001770)
Supplement: S2 Table — (DOCX) [file pbio.3001770.s004.docx]

**S2** **Table. Citations for Data used to Calculate Successional Scores, Ages of Greatest Prevalence, and** *R*_0_ **Values**

| **Disease** | **Citations** |
| --- | --- |
| A. lumbricoides | [1-5] |
| B. pertussis | [6-9] |
| Cryptosporidium spp. | [10-15] |
| DENV | [16-20] |
| Ebola virus | [21-26] |
| ETEC | [27-30] |
| Flavivirus | [19, 31, 32] |
| HAV | [33-37] |
| HBV | [38-42] |
| HCV | [43-46] |
| HIV | [47-51] |
| Influenza | [52-54] |
| Leishmaniasis | [55-59] |
| M. tuberculosis | [49, 60-64] |
| Measles | [65-69] |
| N. americanus | [3, 70-72] |
| N. gonorrheae | [73-75] |
| O. volvulus | [76-79] |
| Plasmodium spp | [80-86] |
| Rotavirus | [87-90] |
| S. pneumoniae | [91-94] |
| Salmonella spp. | [3, 95-98] |
| Schistosoma spp. | [99-104] |
| Shigella | [105-108] |
| T. brucei | [109-113] |
| T. cruzi | [114-119] |
| T. pallidum | [120-126] |
| T. trichiura | [1-3, 127, 128] |
| V. cholerae | [129-133] |
| W. Bancrofti | [134-139] |

**References**

1. Chan M, Guyatt H, Bundy DA, Medley G. The development and validation of an age-structured model for the evaluation of disease control strategies for intestinal helminths. Parasitology. 1994;109(3):389-96.

2. Wiwanitkit V. Intestinal parasitic infections in Thai HIV-infected patients with different immunity status. BMC gastroenterology. 2001;1(1):1.

3. Bethony J, Brooker S, Albonico M, Geiger SM, Loukas A, Diemert D, et al. Soil-transmitted helminth infections: ascariasis, trichuriasis, and hookworm. The Lancet. 2006;367(9521):1521-32.

4. Hagel I, Giusti T. Ascaris lumbricoides: an overview of therapeutic targets. Infectious Disorders-Drug Targets (Formerly Current Drug Targets-Infectious Disorders). 2010;10(5):349-67.

5. Murray PR, Rosenthal KS, Pfaller MA. Medical microbiology: Mosby; 1994.

6. Kretzschmar M, Teunis PF, Pebody RG. Incidence and reproduction numbers of pertussis: estimates from serological and social contact data in five European countries. PLoS Med. 2010;7(6):e1000291.

7. CDC. Pertussis: Epidemiology and Prevention of Vaccine-Preventable Diseases. 13 ed2015.

8. Canada PHAo. Bordetella Pertussis. 2011.

9. WHO. Pertussis. 2016.

10. CDC. Opportunistic Infections. In: Prevention DoHA, editor. 2015.

11. Hunter PR, Nichols G. Epidemiology and clinical features of Cryptosporidium infection in immunocompromised patients. Clinical microbiology reviews. 2002;15(1):145-54.

12. Authority OH. Cryptosporidiosis: Investigative Guidelines. In: Division PH, editor. 2015.

13. Clark DP. New insights into human cryptosporidiosis. Clinical microbiology reviews. 1999;12(4):554-63.

14. Fayer R, Morgan U, Upton SJ. Epidemiology of Cryptosporidium: transmission, detection and identification. International journal for parasitology. 2000;30(12):1305-22.

15. WHO. Guidelines for drinking-water quality: recommendations: World Health Organization; 2004.

16. Mello RFL, Castilho C. A Stuctured Discrete Model for Dengue Fever Infections and the Determination of R_0 from Age-Stratified Serological Data. Bulletin of mathematical biology. 2014;76(6):1288-305.

17. WHO. Dengue and severe dengue. 2016.

18. Kawaguchi I, Sasaki A, Boots M. Why are dengue virus serotypes so distantly related? Enhancement and limiting serotype similarity between dengue virus strains. Proceedings of the Royal Society of London B: Biological Sciences. 2003;270(1530):2241-7.

19. Harrington LC, Scott TW, Lerdthusnee K, Coleman RC, Costero A, Clark GG, et al. Dispersal of the dengue vector Aedes aegypti within and between rural communities. The American Journal of Tropical Medicine and Hygiene. 2005;72(2):209-20.

20. Egger JR. Age and clinical dengue illness. Emerging infectious diseases. 2007;13(6):924-5.

21. Althaus CL. Estimating the Reproduction Number of Ebola Virus (EBOV) During the 2014 Outbreak in West Africa. PLoS Curr. 2014;6. Epub 2015/02/03. doi: 10.1371/currents.outbreaks.91afb5e0f279e7f29e7056095255b288. PubMed PMID: 25642364; PubMed Central PMCID: PMCPMC4169395.

22. Legrand J, Grais RF, Boelle P-Y, Valleron A-J, Flahault A. Understanding the dynamics of Ebola epidemics. Epidemiology and infection. 2007;135(04):610-21.

23. Canada PHAo. Ebolavirus. 2014.

24. Ito H, Watanabe S, Sanchez A, Whitt MA, Kawaoka Y. Mutational analysis of the putative fusion domain of Ebola virus glycoprotein. Journal of virology. 1999;73(10):8907-12.

25. WHO. Ebola virus disease. 2016.

26. Team WER. Ebola virus disease in West Africa—the first 9 months of the epidemic and forward projections. N Engl J Med. 2014;371(16):1481-95.

27. CDC. Enterotoxigenic Escherichia coli (ETEC). 2005.

28. Qadri F, Svennerholm A-M, Faruque A, Sack RB. Enterotoxigenic Escherichia coli in developing countries: epidemiology, microbiology, clinical features, treatment, and prevention. Clinical microbiology reviews. 2005;18(3):465-83.

29. Control CfD, Prevention. Foodborne outbreaks of enterotoxigenic Escherichia coli--Rhode Island and New Hampshire, 1993. MMWR Morbidity and mortality weekly report. 1994;43(5):81, 7.

30. Begum YA, Baby NI, Faruque AS, Jahan N, Cravioto A, Svennerholm A-M, et al. Shift in phenotypic characteristics of enterotoxigenic Escherichia coli (ETEC) isolated from diarrheal patients in Bangladesh. PLoS Negl Trop Dis. 2014;8(7):e3031.

31. WHO. Yellow Fever. 2016.

32. Barnett ED. Yellow fever: epidemiology and prevention. Clin Infect Dis. 2007;44(6):850-6. Epub 2007/02/17. doi: 10.1086/511869. PubMed PMID: 17304460.

33. Gay N, Morgan-Capner P, Wright J, Farrington C, Miller E. Age-specific antibody prevalence to hepatitis A in England: implications for disease control. Epidemiology and infection. 1994;113(1):113-20.

34. CDC. Viral Hepatitis - Hepatitis A Information. 2015.

35. Matheny SC, Kingery JE. Hepatitis A. Am Fam Physician. 2012;86(11):1027-34.

36. Aragonès L, Bosch A, Pintó RM. Hepatitis A virus mutant spectra under the selective pressure of monoclonal antibodies: codon usage constraints limit capsid variability. Journal of virology. 2008;82(4):1688-700.

37. Lees D. Viruses and bivalve shellfish. International journal of food microbiology. 2000;59(1):81-116.

38. Wilson J, Nokes DJ, Carman W. Predictions of the emergence of vaccine-resistant hepatitis B in The Gambia using a mathematical model. Epidemiology and infection. 2000;124(02):295-307.

39. Health ODoP. Acute Hepatitis B. In: Health P, editor. 2012.

40. Chung RT, Kim AY, Polsky B. HIV/Hepatitis B and C co-infection: pathogenic interactions, natural history and therapy. Antiviral chemistry & chemotherapy. 2000;12:73-91.

41. WHO. Hepatitis B Fact Sheet. 2015.

42. Kurashige N, Ohkawa K, Hiramatsu N, Oze T, Yakushijin T, Mochizuki K, et al. Case report Two types of drug-resistant hepatitis B viral strains emerging alternately and their susceptibility to combination therapy with entecavir and adefovir. Antiviral therapy. 2009;14:873-7.

43. WHO. Hepatitis C Fact Sheet. 2015.

44. Sulkowski MS, Mast EE, Seeff LB, Thomas DL. Hepatitis C virus infection as an opportunistic disease in persons infected with human immunodeficiency virus. Clinical Infectious Diseases. 2000;30(Supplement 1):S77-S84.

45. Romano KP, Ali A, Aydin C, Soumana D, Özen A, Deveau LM, et al. The molecular basis of drug resistance against hepatitis C virus NS3/4A protease inhibitors. PLoS Pathog. 2012;8(7):e1002832.

46. CDC. Viral Hepatitis - Hepatitis C Information. 2016.

47. Hollingsworth TD, Anderson RM, Fraser C. HIV-1 transmission, by stage of infection. Journal of Infectious Diseases. 2008;198(5):687-93.

48. Services UDoHaH. Stages of HIV Infection. 2016.

49. Borkow G, Bentwich Z. Eradication of helminthic infections may be essential for successful vaccination against HIV and tuberculosis. Bulletin of the World Health Organization. 2000;78(11):1368-9.

50. Richman DD, Morton SC, Wrin T, Hellmann N, Berry S, Shapiro MF, et al. The prevalence of antiretroviral drug resistance in the United States. Aids. 2004;18(10):1393-401.

51. Ball J, Desselberger U, Whitwell H. Long-lasting viability of HIV after patient's death. Lancet. 1991;338(8758):63. Epub 1991/07/06. doi: 10.1016/0140-6736(91)90063-u. PubMed PMID: 1676124.

52. Fraser C, Donnelly CA, Cauchemez S, Hanage WP, Van Kerkhove MD, Hollingsworth TD, et al. Pandemic potential of a strain of influenza A (H1N1): early findings. Science. 2009;324(5934):1557-61.

53. CDC. Influenza: Epidemiology and Prevention of Vaccine-Preventable Diseases. 13 ed2015.

54. Bean B, Moore B, Sterner B, Peterson L, Gerding D, Balfour H. Survival of influenza viruses on environmental surfaces. Journal of Infectious Diseases. 1982;146(1):47-51.

55. Courtenay O, Quinnell RJ, Garcez LM, Shaw JJ, Dye C. Infectiousness in a cohort of Brazilian dogs: why culling fails to control visceral leishmaniasis in areas of high transmission. Journal of Infectious Diseases. 2002;186(9):1314-20.

56. Chappuis F, Sundar S, Hailu A, Ghalib H, Rijal S, Peeling RW, et al. Visceral leishmaniasis: what are the needs for diagnosis, treatment and control? Nature Reviews Microbiology. 2007;5(11):873-82.

57. Pasquau F, Ena J, Sanchez R, Cuadrado J, Amador C, Flores J, et al. Leishmaniasis as an opportunistic infection in HIV-infected patients: determinants of relapse and mortality in a collaborative study of 228 episodes in a Mediterreanean region. European Journal of Clinical Microbiology and Infectious Diseases. 2005;24(6):411-8.

58. Pearson RD. Leishmaniasis. 2014. In: Merck Manual - Professional Version [Internet]. Available from: <http://www.merckmanuals.com/professional/infectious-diseases/extraintestinal-protozoa/leishmaniasis>.

59. Ferro C, Cárdenas E, Corredor D, Morales A, Munstermann LE. Life cycle and fecundity analysis of Lutzomyia shannoni (Dyar)(Diptera: Psychodidae). Memórias do Instituto Oswaldo Cruz. 1998;93(2):195-9.

60. Sanchez MA, Blower SM. Uncertainty and sensitivity analysis of the basic reproductive rate: tuberculosis as an example. American journal of epidemiology. 1997;145(12):1127-37.

61. Vynnycky E, Fine PE. Lifetime risks, incubation period, and serial interval of tuberculosis. American journal of epidemiology. 2000;152(3):247-63.

62. Wright A, Zignol M, Van Deun A, Falzon D, Gerdes SR, Feldman K, et al. Epidemiology of antituberculosis drug resistance 2002–07: an updated analysis of the Global Project on Anti-Tuberculosis Drug Resistance Surveillance. The Lancet. 2009;373(9678):1861-73.

63. WHO. Tuberculosis Fact Sheet. 2016.

64. Kramer A, Schwebke I, Kampf G. How long do nosocomial pathogens persist on inanimate surfaces? A systematic review. BMC infectious diseases. 2006;6(1):1.

65. Anderson R, May, R.M. Infectious disease of humans. Oxford, UK: Oxford University Press; 1991.

66. Fiebelkorn AP, Goodson JL. Measles (rubeola). CDC Health Information for International Travel 2014: The Yellow Book. 2013:249.

67. Nakano T, Shimono Y, Sugiyama K, NISHIHARA H, HIGASHIGAWA M, KOMADA Y, et al. Clinical features of measles in immunocompromised children. Pediatrics International. 1996;38(3):212-7.

68. Canada PHAo. Measles Virus. 2011.

69. WHO. Measles. 2016.

70. Sabatelli L, Ghani A, Rodrigues L, Hotez P, Brooker S. Modelling heterogeneity and the impact of chemotherapy and vaccination against human hookworm. Journal of The Royal Society Interface. 2008;5(28):1329-41.

71. Canada PHAo. Necator americanus. 2012.

72. Brooker S, Bethony J, Hotez PJ. Human hookworm infection in the 21st century. Advances in parasitology. 2004;58:197-288.

73. Moran JS. Gonorrhoea. BMJ clinical evidence. 2007;2007.

74. Jewett JF. Nonsexual acquisition of genital gonococcal infection. The New England journal of medicine. 1979;301(24):1347.

75. CDC. Gonorrhea - CDC Fact Sheet. 2016.

76. Basáñez Ma-G, Ricárdez-Esquinca J. Models for the population biology and control of human onchocerciasis. Trends in Parasitology. 2001;17(9):430-8.

77. Udall DN. Recent updates on onchocerciasis: diagnosis and treatment. Clinical Infectious Diseases. 2007;44(1):53-60.

78. Onchocerciasis (River Blindness) [Internet]. 2004. Available from: <http://web.stanford.edu/group/parasites/ParaSites2004/Onchocerciasis/>.

79. Simulium venustum (On-line) [Internet]. 2013. Available from: <http://animaldiversity.org/accounts/Simulium_venustum/>.

80. Smith DL, McKenzie FE. Statics and dynamics of malaria infection in Anopheles mosquitoes. Malaria journal. 2004;3(1):13.

81. Victoria SGo. Malaria. In: Services DoHH, editor. 2015.

82. Rowland-Jones SL, Lohman B. Interactions between malaria and HIV infection—an emerging public health problem? Microbes and infection. 2002;4(12):1265-70.

83. Brasil P, de Pina Costa A, Pedro RS, da Silveira Bressan C, da Silva S, Tauil PL, et al. Unexpectedly long incubation period of Plasmodium vivax malaria, in the absence of chemoprophylaxis, in patients diagnosed outside the transmission area in Brazil. Malar J. 2011;10:122.

84. WHO. Malaria. 2014.

85. WHO. Malaria Fact Sheet. 2016.

86. Smith DL, McKenzie FE, Snow RW, Hay SI. Revisiting the basic reproductive number for malaria and its implications for malaria control. PLoS Biol. 2007;5(3):e42.

87. Pitzer VE, Atkins KE, de Blasio BF, Van Effelterre T, Atchison CJ, Harris JP, et al. Direct and indirect effects of rotavirus vaccination: comparing predictions from transmission dynamic models. PLoS One. 2012;7(8):e42320.

88. CDC. Rotavirus: Epidemiology and Prevention of Vaccine-Preventable Diseases. 13 ed2015.

89. Mofenson LM, Brady MT, Danner SP, Dominguez KL, Hazra R, Handelsman E, et al. Guidelines for the Prevention and Treatment of Opportunistic Infections among HIV-exposed and HIV-infected children: recommendations from CDC, the National Institutes of Health, the HIV Medicine Association of the Infectious Diseases Society of America, the Pediatric Infectious Diseases Society, and the American Academy of Pediatrics. MMWR Recommendations and reports: Morbidity and mortality weekly report Recommendations and reports/Centers for Disease Control. 2009;58(RR-11):1.

90. Kirkwood CD. Genetic and antigenic diversity of human rotaviruses: potential impact on vaccination programs. Journal of Infectious Diseases. 2010;202(Supplement 1):S43-S8.

91. Nurhonen M, Cheng AC, Auranen K. Pneumococcal transmission and disease in silico: a microsimulation model of the indirect effects of vaccination. PLoS One. 2013;8(2):e56079.

92. CDC. Pneumococcal Disease (Streptococcus pneumoniae). 2014.

93. Walther BA, Ewald PW. Pathogen survival in the external environment and the evolution of virulence. Biological Reviews. 2004;79(4):849-69.

94. CDC. Pneumococcal Disease: Epidemiology and Prevention of Vaccine-Preventable Diseases. 13 ed2015.

95. Berriman AD, Clancy D, Clough HE, Armstrong D, Christley RM. Effectiveness of simulated interventions in reducing the estimated prevalence of Salmonella in UK pig herds. PLoS One. 2013;8(6):e66054.

96. Victoria SGo. Salmonellosis. In: Services DoHH, editor. 2015.

97. White DG, Zhao S, Sudler R, Ayers S, Friedman S, Chen S, et al. The isolation of antibiotic-resistant Salmonella from retail ground meats. New England Journal of Medicine. 2001;345(16):1147-54.

98. Winfield MD, Groisman EA. Role of nonhost environments in the lifestyles of Salmonella and Escherichia coli. Applied and environmental microbiology. 2003;69(7):3687-94.

99. Woolhouse M, Hasibeder G, Chandiwana S. On estimating the basic reproduction number for Schistosoma haematobium. Tropical Medicine & International Health. 1996;1(4):456-63.

100. Warren KS, Mahmoud A, Cummings P, Murphy DJ, Houser HB. Schistosomiasis mansoni in Yemeni in California: duration of infection, presence of disease, therapeutic management. The American Journal of Tropical Medicine and Hygiene. 1974;23(5):902-9.

101. Secor WE. The effects of schistosomiasis on HIV/AIDS infection, progression and transmission. Current Opinion in HIV and AIDS. 2012;7(3):254-9.

102. CDC. Schistosomiasis FAQs. 2012.

103. Doenhoff MJ, Cioli D, Utzinger J. Praziquantel: mechanisms of action, resistance and new derivatives for schistosomiasis. Current opinion in infectious diseases. 2008;21(6):659-67.

104. Pereira APB, Favre TC, Galvão AF, Beck L, Barbosa CS, Pieri OS. The prevalence of schistosomiasis in school-aged children as an appropriate indicator of its prevalence in the community. Memórias do Instituto Oswaldo Cruz. 2010;105(4):563-9.

105. Joh RI, Hoekstra RM, Barzilay EJ, Bowen A, Mintz ED, Weiss H, et al. Dynamics of shigellosis epidemics: estimating individual-level transmission and reporting rates from national epidemiologic data sets. American journal of epidemiology. 2013:kwt122.

106. Kotloff KL, Winickoff JP, Ivanoff B, Clemens JD, Swerdlow DL, Sansonetti PJ, et al. Global burden of Shigella infections: implications for vaccine development and implementation of control strategies. Bulletin of the World Health Organization. 1999;77(8):651-66.

107. Health ISDo. Shigellosis.

108. Ud-Din AI, Wahid SU, Latif HA, Shahnaij M, Akter M, Azmi IJ, et al. Changing trends in the prevalence of Shigella species: emergence of multi-drug resistant Shigella sonnei biotype g in Bangladesh. PLoS One. 2013;8(12):e82601.

109. Funk S, Nishiura H, Heesterbeek H, Edmunds WJ, Checchi F. Identifying transmission cycles at the human-animal interface: the role of animal reservoirs in maintaining gambiense human african trypanosomiasis. PLoS Comput Biol. 2013;9(1):e1002855.

110. Walker M, Kublin JG, Zunt JR. Parasitic central nervous system infections in immunocompromised hosts: malaria, microsporidiosis, leishmaniasis, and African trypanosomiasis. Clinical infectious diseases: an official publication of the Infectious Diseases Society of America. 2006;42(1):115.

111. WHO. Trypanosomiasis Fact Sheet. 2016.

112. Barrett MP, Vincent IM, Burchmore RJ, Kazibwe AJ, Matovu E. Drug resistance in human African trypanosomiasis. Future microbiology. 2011;6(9):1037-47.

113. Canada PHAo. Trypanosoma brucei - Pathogen Safety Data Sheet 2014.

114. Cordovez JM, Rendon LM, Gonzalez C, Guhl F. Using the basic reproduction number to assess the effects of climate change in the risk of Chagas disease transmission in Colombia. Acta tropica. 2014;129:74-82.

115. CDC. Parasites - African Trypanosomiasis. 2012.

116. Teixeira A, Nitz N, Guimaro M, Gomes C, Santos-Buch C. Chagas disease. Postgraduate medical journal. 2006;82(974):788-98.

117. Rassi A, Marin-Neto JA. Chagas disease. The Lancet. 2010;375(9723):1388-402.

118. Filardi L, Brener Z. Susceptibility and natural resistance of Trypanosoma cruzi strains to drugs used clinically in Chagas disease. Transactions of The Royal Society of Tropical Medicine and Hygiene. 1987;81(5):755-9.

119. dos Santos SMdF, Neto V, Di Pietro A, Almeida BL. [Longevity of Trypanosoma cruzi in the Triatoma infestans]. Revista do Hospital das Clinicas. 1995;51(1):12-4.

120. Aadland D, Finnoff DC, Huang KX. Syphilis Cycles. The BE Journal of Economic Analysis & Policy. 2013;13(1):297-348.

121. Ghanem KG. Syphilis as an opportunistic infection in HIV: THE JOHNS HOPKINS UNIVERSITY; 2009.

122. Cherneskie T. An update and review of the diagnosis and management of syphilis. Region II STD/VIH Prevention Training Center.

123. Stamm LV. Global challenge of antibiotic-resistant Treponema pallidum. Antimicrobial agents and chemotherapy. 2010;54(2):583-9.

124. Turner TB. The preservation of virulent Treponema pallidum and Treponema pertenue in the frozen state; with a note on the preservation of filtrable viruses. The Journal of experimental medicine. 1938;67(1):61-78.

125. CDC. 2012 Sexually Transmitted Diseases Surveillance. 2012.

126. CDC. Syphilis - CDC Fact Sheet. 2016.

127. CDC. Trichuriasis. 2013.

128. Canada PHAo. Trichuris trichiura. 2011.

129. Mukandavire Z, Liao S, Wang J, Gaff H, Smith DL, Morris JG. Estimating the reproductive numbers for the 2008–2009 cholera outbreaks in Zimbabwe. Proceedings of the National Academy of Sciences. 2011;108(21):8767-72.

130. WHO. Cholera Fact Sheet. 2015.

131. Azman AS, Rudolph KE, Cummings DA, Lessler J. The incubation period of cholera: a systematic review. J Infect. 2013;66(5):432-8. Epub 2012/12/04. doi: 10.1016/j.jinf.2012.11.013. PubMed PMID: 23201968; PubMed Central PMCID: PMCPMC3677557.

132. Sjölund-Karlsson M, Reimer A, Folster JP, Walker M, Dahourou GA, Batra DG, et al. Drug-resistance mechanisms in Vibrio cholerae O1 outbreak strain, Haiti, 2010. Emerg Infect Dis. 2011;17(11):2151-4.

133. Chaiyanan S, Chaiyanan S, Huq A, Maugel T, Colwell RR. Viability of the nonculturable Vibrio cholerae O1 and O139. Systematic and Applied Microbiology. 2001;24(3):331-41.

134. Supriatna AK, Anggriani N. Lymphatic filariasis transmission and control: a mathematical modelling approach. Current Topics in Tropical Medicine. 2012:425-42.

135. WHO. Lymphatic filariasis. 2016.

136. Mahanty S, Ravichandran M, Raman U, Jayaraman K, Kumaraswami V, Nutman TB. Regulation of parasite antigen-driven immune responses by interleukin-10 (IL-10) and IL-12 in lymphatic filariasis. Infection and immunity. 1997;65(5):1742-7.

137. Molyneux DH, Bradley M, Hoerauf A, Kyelem D, Taylor MJ. Mass drug treatment for lymphatic filariasis and onchocerciasis. Trends in Parasitology. 2003;19(11):516-22.

138. Lebl K, Brugger K, Rubel F. Predicting Culex pipiens/restuans population dynamics by interval lagged weather data. Parasit Vectors. 2013;6(1):129.

139. USAID. Lymphatic Filariasis. 2016.
