# Supplementary material for: Considering humans as habitat reveals evidence of successional disease ecology among human pathogens
Source: PLoS Biol. 2022 Sep 12;20(9):e3001770. doi: 10.1371/journal.pbio.3001770 (PMC9467372; doi:10.1371/journal.pbio.3001770)
Supplement: S3 Table — (DOCX) [file pbio.3001770.s005.docx]

**Table S3. Rank Values for Infectious Period, Incubation Period, and Duration of Viability Outside the Host**

| 1 | 2 | 3 | 4 | 5 | 6 | 7 | 8 | 9 |
| --- | --- | --- | --- | --- | --- | --- | --- | --- |
| Hours | Days | D/W | Weeks | W/M | Months | M/Y | Years | Decades |
