# Supplementary material for: Considering humans as habitat reveals evidence of successional disease ecology among human pathogens
Source: PLoS Biol. 2022 Sep 12;20(9):e3001770. doi: 10.1371/journal.pbio.3001770 (PMC9467372; doi:10.1371/journal.pbio.3001770)
Supplement: S4 Table — (DOCX) [file pbio.3001770.s006.docx]

**S4 Table. Rank Values for Transmission Distance, Opportunistic, and Mutability**

|  | **0** | **1** | **2** | **3** |
| --- | --- | --- | --- | --- |
| **Distance** |  | Short | Medium | Long |
| **Opportunistic** |  | Yes | ? | No |
| **Mutability** | High | Low |  |  |
