# Supplementary material for: Considering humans as habitat reveals evidence of successional disease ecology among human pathogens
Source: PLoS Biol. 2022 Sep 12;20(9):e3001770. doi: 10.1371/journal.pbio.3001770 (PMC9467372; doi:10.1371/journal.pbio.3001770)
Supplement: S5 Table — (DOCX) [file pbio.3001770.s007.docx]

**Table S5. Categorical Ranks for each Disease used to Calculate Successional Scores**

| Pathogen/  Parasite | Infectious Period | Incubation Period | Pathogen Viability | Distance | Opportunist | Mutability |
| --- | --- | --- | --- | --- | --- | --- |
| A. lumbridcoides | Decades (9) | Months (6) | Decades (9) | Medium (2) | Yes (1) | High (0) |
| B. pertussis | Weeks/ Months (5) | Weeks (4) | Days (2) | Short (1) | Yes (1) | High (0) |
| Cryptosporidium spp. | Days/ Weeks (3) | Days/ Weeks (3) | Weeks/ Months (5) | Medium (2) | No (3) | Low (1) |
| DENV | Days/ Weeks (3) | Days/ Weeks (3) | Months (6) | Long (3) | Yes (1) | Low (1) |
| Ebola virus | Weeks/ Months (5) | Days/ Weeks (3) | Hours (1) | Short (1) | Yes (1) | High (0) |
| ETEC | Days (2) | Days (2) | Months (6) | Medium (2) | ? (2) | Low (1) |
| Flavivirus genus | Weeks (4) | Days (2) | Months (6) | Long (3) | Yes (1) | High (0) |
| HAV | Months (6) | Weeks/ Months (5) | Days (2) | Short (1) | Yes (1) | High (0) |
| HBV | Months/  Years (7) | Months (6) | Weeks (4) | Short (1) | No (3) | Low (1) |
| HCV | Months/  Years (7) | Months (6) | Days (2) | Short (1) | No (3) | Low (1) |
| HIV | Decades (9) | Weeks (4) | Days/ Weeks (3) | Short (1) | No (3) | Low (1) |
| Influenza virus | Days/ Weeks (3) | Days (2) | Hours (1) | Short (1) | Yes (1) | Low (1) |
| Leishmania spp. | Weeks/ Months (5) | Months (6) | Days/ Weeks (3) | Long (3) | No (3) | Low (1) |
| M. tuberculosis | Years (8) | Months (6) | Weeks (4) | Short (1) | No (3) | Low (1) |
| Measles virus | Weeks (4) | Days/ Weeks (3) | Hours (1) | Short (1) | ? (2) | High (0) |
| N. americanus | Years (8) | Months (6) | Weeks (4) | Medium (2) | No (3) | Low (1) |
| N. gonorrhoeae | Weeks/ Months (5) | Days/ Weeks (3) | - | Short (1) | ? (2) | High (0) |
| O. volvulus | Years (8) | Years (8) | Weeks/ Months (5) | Long (3) | ? (2) | High (0) |
| Plasmodium spp. | Years (8) | Days/ Weeks (3) | Weeks/ Months (5) | Long (3) | ? (2) | Low (1) |
| Rotavirus | Days/ Weeks (3) | Days (2) | Weeks/ Months (5) | Medium (2) | ? (2) | Low (1) |
| S. pneumoniae | - | Days (2) | Days (2) | Short (1) | No (3) | Low (1) |
| Salmonella spp. | Days/ Weeks (3) | Days (2) | Weeks (4) | Medium (2) | No (3) | Low (1) |
| Schistosoma spp. | Years (8) | Weeks (4) | Days (2) | Medium (2) | Yes (1) | High (0) |
| Shigella spp. | Weeks (4) | Days (2) | Days (2) | Medium (2) | No (3) | Low (1) |
| T. brucei | Months (6) | Weeks (4) | Hours (1) | Long (3) | Yes (1) | Low (1) |
| T. cruzi | Decades (9) | Weeks (4) | Months/  Years (7) | Long (3) | No (3) | High (0) |
| T. pallidum | Decades (9) | Weeks (4) | - | Short (1) | No (3) | Low (1) |
| T. trichiura | Years (8) | Months (6) | Weeks/ Months (5) | Medium (2) | Yes (1) | Low (1) |
| V. cholerae | Weeks (4) | Days (2) | Years (8) | Medium (2) | ? (2) | Low (1) |
| W. bancrofti | Years (8) | Months/  Years (7) | Weeks (4) | Long (3) | Yes (1) | High (0) |
